# A Case of* De Novo* CD5+ Disseminated Intravascular Large B-Cell Lymphoma Presenting as Multiorgan Failure

**DOI:** 10.1155/2016/6239416

**Published:** 2016-09-29

**Authors:** Daulath Singh, Devika Kapuria, Suparna Nanua, Rakesh Gaur

**Affiliations:** ^1^Department of Internal Medicine, The University of Missouri Kansas City School of Medicine, 2301 Holmes Street, Kansas City, MO 64108, USA; ^2^Department of Laboratory Medicine, Saint Luke's Hospital of Kansas City, 4401 Wornall Road, Kansas City, MO 64111, USA; ^3^Department of Hematology/Oncology, Saint Luke's Hospital of Kansas City, Saint Luke's Cancer Specialists, 4321 Washington Street, No. 4000, Kansas City, MO 64111, USA

## Abstract

Intravascular large B-cell lymphoma is an extremely rare extranodal lymphoma that proliferates in the lumen of the blood vessels while sparing the organ parenchyma. It usually presents with CNS and skin involvement. A 65-year-old Caucasian female presented with fevers and chills of 3-4 months' duration. Bone marrow biopsy done 3 months prior showed no significant myelodysplasia or lymphoid aggregates. The patient later died due to multiorgan failure. A bone marrow biopsy showed 20–30% CD5+ B cells consistent with infiltrative large B-cell lymphoma. An autopsy performed revealed diffuse intravascular invasion by lymphoma cells. Multiorgan involvement by intravascular B-cell lymphoma is very rare. Based on our literature review and to the best of our knowledge, there are only 5 case reports describing the presentation of this lymphoma with multiorgan failure. The immunophenotypic studies performed revealed that our patient had* de novo* CD5+ intravascular large B-cell lymphoma which is known to be aggressive with very poor prognosis. Although it is an extremely rare lymphoma, it should be considered as a potential cause of multiorgan failure when no other cause has been identified. A prompt tissue diagnosis and high-dose chemotherapy followed by ASCT can sometimes achieve remission.

## 1. Introduction 

Intravascular large B-cell lymphoma is an extremely rare extranodal lymphoma, proliferating in the lumen of the blood vessels, particularly small vessels, and capillaries, while the parenchyma of the involved organs is spared [1]. Median age at diagnosis is sixth to seventh decade with no sex predilection [2, 3].

The clinical presentation is varied and often includes symptoms related to organ dysfunction caused by occlusion of blood vessels. Constitutional B symptoms (i.e., fever, night sweats, and weight loss) are seen in the majority of patients (55 to 85 percent) [2, 3]. Patients most commonly present with symptoms related to the involvement of the central nervous system and skin. Others present with involvement of the bone marrow (75 percent), spleen (67 percent), and liver (55 percent) [3].

Here we have a case of* de novo* disseminated intravascular large B-cell lymphoma presenting as multiorgan dysfunction. The first case of IVL presenting as multiorgan failure was described by Deusch et al. in 1999 [4]. Based on our literature review, there is a total of 5 case reports so far describing the presentation of IVL as MODS.

## 2. Case Report

65-year-old Caucasian female with the history of ulcerative colitis and migraine presented with chief complaints of fevers and chills of 3-4 months' duration associated with diaphoresis, fatigue, and dyspnea. She visited an urgent care clinic where she was given antibiotics and albuterol for presumed pneumonia. Her symptom worsened after 5 days of antibiotics and was referred to ED.

In the ED, CT angiography chest showed tiny nonocclusive pulmonary embolism in the right lower lobe of the lung with moderate bilateral pleural effusions. Treatment was started with heparin drip and diagnostic thoracentesis was done. Pleural fluid was indicative of transudate with negative cytology. The initial physical exam did not reveal skin rashes, palpable hepatosplenomegaly, lymphadenopathy, or focal neurological deficits. Cardiology was consulted over the hospital course for worsening dyspnea. 2D ECHO showed hyperdynamic circulation with LVEF 70%, normal right heart function, and no valve abnormalities. Cardiac nuclear perfusion stress test was negative for coronary artery disease.

Laboratory work upon admission showed a hemoglobin of 8.4 g/dL with normal WBC and platelet counts, low albumin (2.4 mg/dL), elevated alkaline phosphatase (400 IU/L), and serum creatinine (1.4 mg/dL). Hematology/Oncology Team was consulted for anemia. Anemia workup was negative for hemolysis. Of note, she had a bone marrow biopsy done 3 months prior to admission for evaluation of anemia, which revealed hypocellular marrow, low iron stores, and absence of significant myelodysplasia or abnormal lymphoid aggregates with unremarkable flow cytometric findings.

The patient developed jaundice after RBC transfusion with elevated bilirubin (8 mg/dL) and high LDH (1795) (normal 313–618 IU/L). CT abdomen showed hepatosplenomegaly, femoral vein thrombus, and nonspecific colitis. On day 8 of admission, she became critically ill with shock, severe lactic acidosis, and liver/renal/respiratory failure. Lab studies showed INR of 5.4, hemoglobin of 7 gm/dL, and platelet count of 139 TH/uL. Peripheral blood smear picture was consistent with a myelophthisis process and flow cytometry showed a CD5+ clonal large B-cell population. With the possibility of lymphoma, high-dose steroids were started. On day 12, she died due to severe shock, refractory acidosis, severe coagulopathy, and MODS.

**Figure 1:**
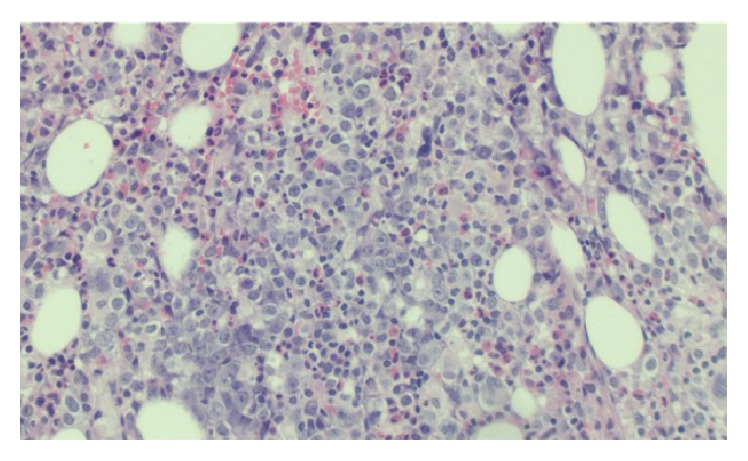
Bone marrow core biopsy 100x showing atypical large cells.

**Figure 2:**
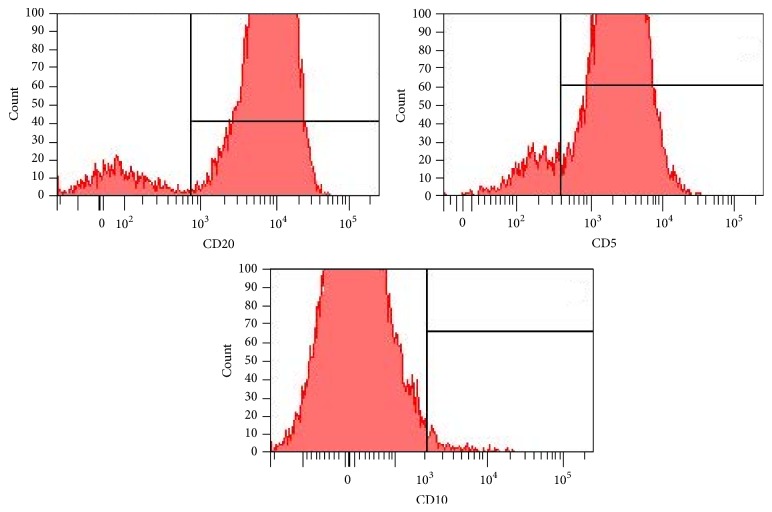
Bone marrow flow cytometry analysis showing CD5+ and CD10− large B cells.

Repeat bone marrow evaluation revealed a hypercellular marrow with 20–30% CD5+ clonal large B-cell population consistent with marrow infiltration with large B-cell non-Hodgkin's lymphoma (Figures 1 and 2). These B cells were positive for CD19, CD20, CD23, and CD52 with coexpression of CD5 and monotypic expression of immunoglobulin lambda surface light chains. B cells were negative for cyclin D1. There was evidence of rare hemophagocytosis noted on the marrow aspirate. Additional workup was negative for PNH, JAK2 mutation, and BCR/ABL1 translocation. Autoimmune and infectious workup including hepatitis came back negative.

An autopsy performed showed disseminated diffuse large B-cell lymphoma. Microscopic analysis of solid organs including heart, lungs, kidneys, intestines, spleen, pancreas, liver, urinary bladder, and adrenal glands revealed diffuse intravascular invasion of major/medium/small vessels by an infiltrate consisting of neoplastic large B cell. Disseminated intravascular coagulopathy also noted on the autopsy was most likely due to thrombosis from the lymphoma cells.

## 3. Discussion 

Intravascular lymphomatosis was first described by Pfleger and Tappeiner in 1959 as angioendotheliomatosis proliferans systemisata [5]. They postulated that the tumor cells were of endothelial origin, but subsequent immune histochemical studies confirmed the lymphoid nature of the neoplasm. IVL is a form of non-Hodgkin's lymphoma of mostly B-cell type and less commonly of T or natural killer cell type [2, 6]. WHO classification of hematopoietic tumors defines IVLBCL as an extranodal diffuse large B-cell lymphoma characterized by the presence of neoplastic lymphocytes only in the lumina of the blood vessels, particularly capillaries [7].

Clinical presentation is extremely heterogeneous, ranging from monosymptomatic forms, such as fever, pain, or local symptoms, to the combination of B symptoms (fevers, weight loss, and night sweats) and rapidly progressing manifestations of multiorgan failure [2]. This is the reason why the majority of cases are diagnosed postmortem [1]. Based on the different clinical features, two variants of IVL have been described—Asian and Western variants. Western variant most commonly presents with CNS and skin involvement. Asian variant commonly presents with bone marrow, spleen, and liver involvement [2]. IVLBCL usually arises without tumor masses or LAD, both in Japanese and in Western variants [2, 8]. Intravascular nature of the tumor could be explained by two factors: (1) tumor cells lack CD29 (*β*1 integrin) and CD54 (intercellular adhesion molecule- (ICAM-) 1), which are essential for lymphocyte homing and transvascular migration; (2) aberrant expression of CD11a and CD49d (very late antigen- (VLA-) 4) on tumor cells enables them to stay in the luminal space via attachment to endothelial cells. Thus, parenchymal and bone marrow infiltration by tumor cells is not recognized until the progressive stage making antemortem diagnosis of IVLBCL difficult [9].

Laboratory features commonly include anemia in nearly 65% of patients. Increased serum lactate dehydrogenase and beta 2-microglobulin levels are observed in more than 80% to 90% of patients [2]. Also, elevated ESR and sometimes abnormal hepatic, renal, and thyroid functions are observed. Serum IL-2 receptor levels are also a sensitive marker of lymphoma [10]. Prostatic acid phosphatase, despite having low specificity, has been proposed as a possible tumor marker of IVL [11].

Diagnosis of IVLBCL is made by demonstration of the presence of large lymphoma cells within small-to-medium blood vessels of major organs including skin [7]. The cutaneous manifestation is variable, presenting as erythema, purpura, telangiectasia with or without swelling, tenderness, or hotness, which all wax and wane. Proliferated lymphoma cells occlude capillary vessels that activate coagulation cascade and thrombi are formed within the vessel Lumina. If the invasion is not sufficient to cause occlusion of the vessels, skin lesion does not occur. Therefore, the lack of a skin lesion does not necessarily mean the absence of invasion of lymphoma cells in the skin [12]. However, random skin biopsies have now become one of the common methods of diagnosing IVBCL, both from normal and from lesioned skin including senile hemangiomas [13]. As reported in multiple case reports and literature reviews, random skin biopsies often demonstrate lymphoma cells in the vessels of deep dermis and subcutaneous tissues. In order to yield positive results, the biopsy should include the dermis as well as the deeper layers, together with the hypodermic adipose tissue, the sample should be relatively large and the procedure should be performed at more than three different locations, including the upper arm, thigh, and the abdomen [14]. It has been suggested that the more the random skin biopsies, the more accurate the diagnosis of IVBCL [15]. In the case series by Arai et al., a total of 32 biopsy specimens collected from the 3 cases indicated that 16 of the 17 sites on the lesional skin and 1 of the 15 sites on the healthy-looking skin were positive for neoplastic cells. This finding suggested that IVL cells occurred more frequently in the lesional skin than in the healthy-looking skin [16]. Antemortem diagnosis of this lymphoma is challenging because there are no pathognomonic features or markers of IVL. Furthermore, because of the intravascular progression of the neoplastic lymphoid cells and the tendency to mimicking other diseases, accurate and definitive diagnosis of IVL has been considered to be difficult [17]. Although IVL is a clonal proliferation of lymphocytes, it is uncommon to find significant adenopathy, organomegaly, or circulating cells in peripheral blood smear, bone marrow, or CSF [18].

Malignant cells uniformly express pan-B-cell antigens (CD20, CD79a) and variably express other antigens such as CD5 (38%) and CD10 (13%) [19]. IVBCL can arise* de novo* and can be secondary to other tumors like SLL, follicular lymphoma, or MALToma [6]. In our case, the B cells were positive for CD5, CD20, and CD23 and negative for cyclin D1. It is unlikely that the IVL was a transformed CLL/SLL as she had no lymphadenopathy, lymphocytosis, and a negative bone marrow evaluation 3 months prior to presentation. The possibility of transformation from mantle cell lymphoma is low due to positive CD23 and negative cyclin D1 expression. The unique feature of our case was the presence of* de novo* CD5 positive large cell lymphoma which has been described as a distinct entity with a very aggressive clinical course [20]. In the study from Murase et al., it was noted that CD5+ tumors were significantly associated with higher frequencies of thrombocytopenia and bone marrow/peripheral blood involvement and lower frequencies of neurologic abnormalities as compared to CD5− tumors [3]. There was no significant difference found in the prognosis between CD5+ and CD5− IVBCL patients in the same study.

The case series by Ferreri et al., 2004, had 80% cases undergoing treatment with CHOP or CHOP-like regimen with around 1/3 patients surviving at the end of 3 years. Two of the 4 patients who underwent autologous stem cell transplant (ASCT) after consolidation with chemotherapy survived. Half of the patients with IVLBCL treated with anthracycline-based chemotherapy relapsed (median time to progression, 7 months) and died within 18 months from diagnosis [7]. Case series by Bouzani et al., 2006, showed a combination of anthracycline-based chemotherapy and rituximab produced durable remissions and prolonged survival for patients with this rare and aggressive disease.

The cutaneous variant of IVLBCL described, mostly, involves women. This variant has a much better outcome and prognosis than disseminated forms. However, therapeutic outcome differed between patients with single and multiple cutaneous lesions, with worse outcome in the multiple cutaneous variants.

All patients diagnosed with IVLBCL at the time of diagnosis are considered disseminated. R-CHOP plus CNS prophylaxis is considered the treatment of choice in these patients as the lymphoma is advanced at the time of diagnosis [21]. The majority of patients with CNS disease treated with anthracycline-based chemotherapy will relapse and die within 9 months of diagnosis [22]. CNS prophylaxis is strongly recommended because CNS recurrence at 3 years is still as high as 25% [23–25]. The fact that several patients have CNS involvement at diagnosis or relapse supports the recommendation to use chemotherapy combinations containing drugs with high CNS bioavailability such as high-dose MTX and high-dose cytarabine in these patients. Experience with ASCT is limited, but it has been used to successfully treat few intravascular lymphoma cases [2, 3, 7].

Our case was unique due to the* de novo *nature of the lymphoma and CD5 positivity of the large B cells. Although it is an extremely rare lymphoma, it should be considered as a potential cause of multiorgan dysfunction when no other cause has been identified. The diagnosis needs a high index of suspicion, especially in the background of relevant clinical, laboratory, and tissue pathologic findings.

## Abbreviations

CNS: Central nervous system
CT: Computed tomography
LDH: Lactate dehydrogenase
ASCT: Autologous hematopoietic stem cell transplant
IVL: Intravascular lymphoma
IVLBCL: Intravascular large B-cell lymphoma
MODS: Multiorgan dysfunction syndrome
LVEF: Left ventricular ejection fraction
PNH: Paroxysmal nocturnal hemoglobinuria
LAD: Lymphadenopathy
ESR: Erythrocyte sedimentation rate
CLL: Chronic lymphocytic leukemia
SLL: Small lymphocytic lymphoma
MTX: Methotrexate.
